# Estimating the prevalence of alcohol abuse using phosphatidylethanol

**DOI:** 10.1192/j.eurpsy.2023.619

**Published:** 2023-07-19

**Authors:** Y. Razvodovsky

**Affiliations:** Institute of Biochemistry National Academy of Science, Grodno, Belarus

## Abstract

**Introduction:**

Currently, there is an active development of methods for the laboratory diagnosis of alcohol abuse using biochemical markers.

**Objectives:**

The aim of this work was to estimate the prevalence of alcohol abuse among the urban population of Belarus using the concentration of phosphatidylethanol in the blood as a biochemical marker of alcoholism.

**Methods:**

220 blood samples from Grodno residents of both sexes aged 15 to 65 were analyzed. The AUDIT questionnaire was used as a screening tool. Determination of the concentration of phosphatidylethanol in the blood was carried out using the method of high performance liquid chromatography - tandem mass spectrometry (HPLC - MS).

**Results:**

The average concentration of phosphatidylethanol in the blood of men and women was 266.11±54.57 and 55.27±9.43 nmol/ml, respectively. In 9.6% of blood samples, the concentration of phosphatidylethanol exceeded the threshold level of alcohol abuse. It was found that the concentration of phosphatidylethanol in the blood does not correlate with the total score, as well as the frequency and quantitative characteristics of the AUDIT screening test.

**Conclusions:**

Determining the concentration of phosphatidylethanol in the blood is a more reliable way to diagnose alcohol abuse than using screening tools.

**Disclosure of Interest:**

None Declared

